# Supplementary material for: Machine learning based classification of aggressive and malignant renal tumors from multimodal data
Source: PLOS Digit Health. 2026 Feb 20;5(2):e0001225. doi: 10.1371/journal.pdig.0001225 (PMC12923042; doi:10.1371/journal.pdig.0001225)
Supplement: S1 Appendix — (DOCX) [file pdig.0001225.s001.docx]

**S1 Appendix Representation learning**

In our application of SimCLR, we use the following augmentations using torchvison.transforms: vertical flip, uniformly sampled rotation from -5 to 5 degrees, scaling with a scaling factor uniformly sampled from an interval of 0.9 to 1.1, Gaussian blur with a kernel size of 25, and additive Gaussian noise (zero mean and standard deviation of 0.1) multiplied with a Bernoulli RV with p = 0.5. For the network, we use ResNet-18 with four input channels for the encoder and a simple MLP with 128 neurons followed by the ReLU activation function for the projection head.
