# Supplementary material for: Machine learning based classification of aggressive and malignant renal tumors from multimodal data
Source: PLOS Digit Health. 2026 Feb 20;5(2):e0001225. doi: 10.1371/journal.pdig.0001225 (PMC12923042; doi:10.1371/journal.pdig.0001225)
Supplement: S2 Appendix — (DOCX) [file pdig.0001225.s002.docx]

**S2 Appendix Model Architecture**

In this section, we describe the architecture of the models employed in this study. We used Python, scikit-learn, and PyTorch to train and evaluate the models. In the notation used below “+” represents the concatenation of vectors, “-” represents the subsequent layer of a network, RF refers to the random forest algorithm, FC layer (I, J) is a fully connected layer with weights and biases and with an input vector of I components and an output vector of J components, ReLU refers to component-wise ReLU activation, and Softmax refers to the softmax activation.

- Model 1: Image Features (512) - FC Layer (512, 3) - Softmax - Class Probabilities (3).
- Model 2: Clinical Features (16) - RF - Class Probabilities (3).
- Model 3: Clinical Features (9) - RF - Class Probabilities (3).
- Model 4: Clinical Features (16) + Tumor Size (1) - RF - Class Probabilities (3).
- Model 5: Clinical Features (9) + Tumor Size (1) - RF - Class Probabilities (3).
- Model 6: Image Features (512) + Clinical Features (9) + Tumor Size (1) - RF - Class Probabilities (3).
- Model 7: Image Features (512) - FC Layer (512, 10) - ReLU + Clinical Features (9) + Image size (1) - FC Layer (20, 3) - Softmax - Class Probabilities (3).
- Model 8: Image Features (20) + Clinical Features (9) + Image Size (1) - RF - Class Probabilities (3).
- Model 9: Image Features (20) + Clinical Features (9) + Tumor Size (1) - FC Layer (20, 3) - Softmax - Class Probabilities (3).

All SimCLR embeddings, the trained SimCLR model, fold splits, and the code used for training are available in the following GitHub repository: https://github.com/mehrnegara/renal_mass_classification
