# Supplementary material for: Machine learning based classification of aggressive and malignant renal tumors from multimodal data
Source: PLOS Digit Health. 2026 Feb 20;5(2):e0001225. doi: 10.1371/journal.pdig.0001225 (PMC12923042; doi:10.1371/journal.pdig.0001225)
Supplement: S3 Appendix — (DOCX) [file pdig.0001225.s003.docx]

**S3 Appendix Nested Five-Fold Cross-Validation**

We use nested five-fold cross-validation for hyperparameter tuning and model evaluation. This process begins by splitting data into two sets: a training and validation set (80%) and a test set (20%). Next, we perform five-fold cross-validation on the training and validation set to identify the optimal hyperparameters. This involves splitting this set into 5 folds, using each fold for validation while training on the remaining folds. The optimal hyperparameters identified are retained, and the model is trained using the entire training and validation set. Thereafter, we evaluate the trained model on the test set, which remains unseen to the network during the hyperparameter tuning process. We repeat this procedure for five distinct non-overlapping test sets to find probability predictions for each subject. Finally, we use these probabilities to construct a receiver operating characteristic curve and compute the area under curve (AUC) for each model. Further details are provided below.

**Hyperparameters, search ranges/criteria**

This process constitutes a nested five-fold cross-validation, where the outer loop provides unbiased model evaluation and the inner loop performs hyperparameter tuning within the training/validation data.

For random forests, we used randomized search with 20 iterations per fold, and the best hyperparameters were selected based on the average ROC-AUC (One-vs-Rest) across the validation folds. Since hyperparameter optimization was performed independently within each fold of the nested five-fold cross-validation, the best hyperparameters were not the same across folds. For this reason, we report the search space and selection criterion (Table A1) rather than listing the specific hyperparameter values for each fold for random forest models.

**Table A1. Hyperparameter search space used for Random Forest models in the nested five-fold cross-validation. The best hyperparameters were selected based on average ROC-AUC (One-vs-Rest) across the validation folds.**

| **Hyperparameter** | **Search Range / Options** |
| --- | --- |
| Number of trees | 100–500 |
| Maximum depth | Unlimited |
| Maximum features per split | 1 to √(number of input features) |
| Minimum samples to split a node | 2–10 |
| Minimum samples per leaf | 1–10 |
| Bootstrap sampling | Yes / No |
| Splitting criterion | Gini impurity |

For the SimCLR pretraining, we based our implementation on the reference PyTorch code available at https://github.com/sthalles/SimCLR.We explored several augmentation configurations (e.g., varying scaling ranges and inclusion/exclusion of specific transformations) and selected the final setup based on downstream classification validation performance.

For the MLP classifier, we used fixed hyperparameters without performing a grid or randomized search; the learning rate, regularization strength, and batch size followed the reference implementation. These settings were chosen for stability and to maintain consistency with the SimCLR pretraining framework. The hyperparameters for MLP and SimCLR pretraining are consolidated in Table A2.

**Table A2. Hyperparameters for the MLP classifier and SimCLR pretraining.**

| **Component** | **Hyperparameter** | **Value / Range** | **Notes** |
| --- | --- | --- | --- |
| MLP Classifier | Learning rate | 3 × 10⁻⁴ | Fixed |
|  | L2 regularization | 8 × 10⁻⁴ | Fixed |
|  | Batch size | 256 | Fixed |
|  | Epochs | 1000 (model 1, model 7), 400 (model 7) | Fixed |
|  | Weight initialization | PyTorch default | Fixed |
| SimCLR Pretraining | Encoder | ResNet-18, 4 input channels | Fixed |
|  | Projection head | 1 hidden layer: Linear (512 → 512) + ReLU; Output layer: Linear (512 → 128) | Hidden layer followed by output layer projecting to 128; matches reference repo |
|  | Embedding dimension | 512 | Default from repo |
|  | Output dimension | 128 | Default from repo |
|  | Temperature | 0.07 | Default from repo |
|  | Number of views | 2 | Default from repo |
|  | Batch size | 224 | Fixed |
|  | Learning rate | 3 × 10⁻⁴ | Default from repo |
|  | Epochs | 1000 | Fixed |
|  | Weight decay | 8 × 10⁻⁴ | Default from repo |
|  | Augmentations | Vertical flip; rotation ±5°; affine scaling 0.9–1.1; Gaussian blur (kernel = 0.1 × image size); additive Gaussian noise σ = 0.1, applied with probability 0.5 | Empirically tuned based on downstream validation performance |
|  | Reference implementation | [SimCLR PyTorch](https://github.com/sthalles/SimCLR) | Used as baseline for defaults |

**Data leakage**

Feature extraction from images using SimCLR and PCA is an unsupervised process that does not use label information. The encoder is trained independently of the downstream classification folds, so no information from the test sets is used. As a result, this step does not introduce data leakage into the classification models.

Min-max normalization was applied to all features across the dataset. While this meant that the test fold values also contributed to the scaling parameters, no label information was used in this process, and all downstream evaluations were performed on held-out patient data. We note that in practice, the scaling introduces minimal bias since the feature ranges were similar across patients. For CT images, clipping values were used for min-max normalization. This transformation is independent of the data splits and does not introduce any leakage between training and test folds.

Feature importances were computed from the feature_importances_ attribute of RandomForestClassifier in scikit-learn within a nested cross-validation framework (5 outer folds × 5 inner folds). For each outer fold, the model was trained on the full outer training set using the best hyperparameters selected via inner validation folds. Importances were extracted using the mean decrease in impurity and averaged across all outer folds. The resulting values reflect the relative contribution of each feature based on the training data, not the held-out test sets.

**Patient-level split details**

All data splits were performed at the patient level to ensure that no patient contributed samples to both training and test sets. A nested five-fold cross-validation procedure was used: in the outer loop, data were divided into five non-overlapping test sets, each containing 20% of patients, with the remaining 80% used for training and validation. Within each outer fold, a five-fold cross-validation was performed on the training/validation set to select optimal hyperparameters, ensuring that each inner fold also contained distinct patients for training and validation. After hyperparameter selection, the model was retrained on the entire training/validation set and evaluated on the held-out test set. This process was repeated for all five outer folds to generate unbiased, patient-level probability predictions for downstream analysis.

All SimCLR embeddings, trained SimCLR model, fold splits and corresponding labels, and code for classifier training are available at [https://github.com/mehrnegara/renal_mass_classification]. Experiments were conducted with a fixed random seed (123457). Training the self-supervised model to get the representation of the 4-phase CECT images on an NVIDIA Tesla P100 GPU took approximately 2 hours, and classifier training on an NVIDIA A40 GPU (96 GB memory, 4 CPUs per task, 1 node) took about 40 minutes.
