# Supplementary material for: Machine learning based classification of aggressive and malignant renal tumors from multimodal data
Source: PLOS Digit Health. 2026 Feb 20;5(2):e0001225. doi: 10.1371/journal.pdig.0001225 (PMC12923042; doi:10.1371/journal.pdig.0001225)
Supplement: S1 Table — (DOCX) [file pdig.0001225.s004.docx]

**S1 Table. Histologic classification of renal masses**

| **Benign** | **Malignant & Indolent** | **Malignant & Aggressive** |
| --- | --- | --- |
| Oncocytoma  Papillary adenoma  Metanephric adenoma  Non-epithelioid angiomyolipoma  Other benign renal masses | Indolent ccRCC  Indolent papillary RCC  Clear-cell papillary RCC  Chromophobe RCC  Tubulocystic RCC  Mucinous tubular and spindle cell RCC  Succinyl dehydrogenase- deficient RCC  Epithelioid angiomyolipoma | Aggressive ccRCC  Aggressive papillary RCC  Collecting duct RCC  Translocation-associated RCC  Hereditary leiomyomatosis RCC  Unclassified RCC  Other malignant non-RCC tumors |

ccRCC = Clear Cell Renal Cell Carcinoma, RCC = Renal Cell Carcinoma
